# Migratory connectivity in the Loggerhead Shrike (*Lanius ludovicianus*)

**DOI:** 10.1002/ece3.4415

**Published:** 2018-10-24

**Authors:** Amy A. Chabot, Keith A. Hobson, Steven L. Van Wilgenburg, Guillermo E. Pérez, Stephen C. Lougheed

**Affiliations:** ^1^ Department of Biology Queen's University Kingston Ontario; ^2^ Environment and Climate Change Canada Saskatoon Saskatchewan; ^3^ Global Alliance for Animals and People Valdivia Chile

**Keywords:** deuterium, differential migration, leap‐frog migration, microsatellites, migratory connectivity, stable isotopes

## Abstract

**Aim:**

We combine genetic and stable isotope data to quantify migration patterns in Loggerhead Shrike (*Lanius ludovicianus*), a species of conservation concern in North America, to assess how connectivity differs and impacts population evolution, ecology, and conservation.

**Location:**

We sampled shrikes across the majority of their nonbreeding range, from the Atlantic Coast to the western United States east of the Rocky Mountains and throughout Mexico.

**Methods:**

Our study used a Bayesian framework using δ^2^H_f_ from a breeding season origin feather and nuclear genetic microsatellite markers to distinguish between co‐occurring migratory and nonmigratory individuals on the wintering grounds and, for migrants, to assign individuals to a breeding ground origin and genetic group.

**Results:**

Migratory shrikes were present throughout the nonbreeding range but the proportion differed among sample areas. Four main wintering areas were identified. Connectivity ranged from weakly negative in birds wintering on the Atlantic Coast to strongly positive between wintering grounds in the southwestern United States and Mexico and northwestern breeding populations. Connectivity was weakest in *L. l. migrans*, and strongest in *L. l. mexicanus* and *L. l. excubitorides*. Although believed to be nonmigratory, long‐distance movements of individuals were observed in *L. ludovicianus* and *L. l. mexicanus*. Our data support a pattern of chain migration, again most notable in the western half of the species nonbreeding range, and differential migration based on age.

**Main conclusions:**

Our study provides of one such of the first quantitative measures of migratory connectivity and is among the first studies of a short‐distance migratory passerine in North America. The higher migratory connectivity among western, versus eastern populations, and less severe population declines attributable to habitat loss or reproductive success, may result in more localized and/or less severe limiting factors for western populations and more severe on the Atlantic coast and Mississippi Alluvial Valley wintering grounds.

## 1 INTRODUCTION

Throughout the world, nearly one‐fifth of bird species migrate between separate breeding and wintering areas, with the proportion of migratory species tending to increase with increasing distance from the equator (Somveille, Manica, Butchart, & Rodrigues, 2013). These species encounter a variety of conditions and threats throughout the year because of their seasonal movements. A broad range of migratory behavior and patterns exist, which likely have differential costs and benefits among populations and even cohorts therein and likely evolved to serve as a means of reducing competition for resources (Gauthreaux, 1982; Holmgren & Lundberg, 1993; Ketterson & Nolan, 1983; Lundberg & Alerstam, 1986).

Migratory connectivity, as defined by Webster, Marra, Haig, Bensch, and Holmes (2002), is the extent to which individuals from the same breeding area migrate to the same stopover sites or wintering areas and vice versa. Connectivity can vary from weak, as when populations from the same breeding area occupy many different wintering areas, to strong, when populations from the same breeding area overwinter in the same wintering area (Webster et al., 2002). The strength of migratory connections is the result of both evolutionary and ecological processes (Marra, Norris, Haig, Webster, & Royle, 2006; Webster et al., 2002), and we are only just beginning to understand the factors that shape inter‐ and intraspecific differences in migratory patterns and their implications (Elser, 2000; Marra, Hunter, & Perrault, 2011; Runge, Martin, Possingham, Willis, & Fuller, 2014; Webster & Marra, 2005). As migratory connectivity is an important determinant of population demography, conservation management actions also require that these connections be quantified (Faaborg et al., 2010; Hostetler, Sillett, & Marra, 2015; Martin et al., 2007; Runge et al., 2014; Taylor & Norris, 2010).

Despite its apparently recent description (Webster et al., 2002), biologists noted the patterns of connectivity of individuals in nonbreeding areas long before the term “migratory connectivity” was coined (e.g., Nilsson, 1858 in Alerstam & Hedenström, 1998; Salomonsen, 1955). Salomonsen (1955), in a review of the spatial segregation of wintering populations of numerous bird species, described populations that mixed freely within a winter area (i.e., displayed weak connectivity) as exhibiting synhiemy. Conversely, populations with distinct wintering grounds exhibited allohiemy, which could be grouped generally into one of four patterns (Salomonsen, 1955): (a) leap‐frog migration encompasses the situation wherein the latitudinal sequence of different populations’ wintering areas is the reverse of their breeding areas, (b) cross‐wise migration occurs when the migration routes of neighboring populations cross during migration, (c) parallel migration occurs when adjacent populations breeding at the same latitude but different longitudes migrate parallel to each other and, (d) longitudinal migration occurs when populations migrate along the same longitude but settle at different latitudes, as in chain migration where the winter quarters of different populations are situated in the same latitudinal sequence as during the breeding season (Nilsson, 1858 in Alerstam & Hedenström, 1998). Even in the absence of population‐level differences in migratory behavior, differential migration, in which birds of differing age or sex segregate on the wintering grounds, can create similar patterns.

Migration can act both directly and indirectly on survival and reproduction (Faaborg et al., 2010; Marra et al., 2011; Sillett & Holmes, 2002). For example, factors occurring on the wintering grounds can have carryover effects on phenology (Marra, Hobson, & Holmes, 1998; McKellar, Marra, Hannon, Studds, & Ratcliffe, 2013) or reproductive success (Norris, Marra, Kyser, Sherry, & Ratcliffe, 2004), and population trends have even been correlated with migratory strategies within populations (Gilroy, Gill, Butchart, Jones, & Franco, 2016). Until recently, our understanding of these impacts remained largely theoretical. A surge in research on both topics over the last two decades, focused mainly on birds, has resulted from major advances in techniques for studying the movements of animals (reviewed in Hobson and Norris 2008).

The Loggerhead Shrike (*Lanius ludovicianus*) is one of only two species of shrikes (Order Passeriformes) that occur in North America, and the only species of “true shrike” (Subfamily Laniinae, Family Laniidae) endemic to the continent. The species breeding range extends from southern Canada throughout the United States and southern Mexico. Shrikes breeding above 40°N are generally obligate migrants (Yosef, 1996; Burnside 1987), while nonmigratory individuals are thought to maintain a territory year‐round (Yosef, 1996). With the exception of the Gulf Coast region in northern Mexico, where the species is believed to occur only in the nonbreeding season (Yosef, 1996), the wintering range of migratory Loggerhead Shrikes entirely overlaps that of nonmigratory conspecifics (Yosef, 1996).

North American Breeding Bird Survey (BBS) data (Sauer et al., 2017) indicate significant (3.18% year^−1^) and range‐wide population declines in Loggerhead Shrike since the inception of the BBS in the 1960s, and it has been identified by the North American Bird Conservation Initiative as a “Common Bird in Steep Decline” (Berlanga et al., 2010). Habitat loss due to succession and human development likely contributed to the initial declines (Cade and Woods 1997, Pruitt, 2000), but continued population declines are outpacing habitat loss in the breeding season suggesting other limiting factors (Pruitt, 2000). Migratory populations have experienced more persistent and drastic declines than nonmigratory conspecifics (Sauer et al., 2017), highlighting the need to quantify migratory connectivity and migration patterns such that limiting factors on stopover sites and on the wintering grounds can be identified and addressed (Pruitt, 2000; Tischendorf, 2015). To date, research on the wintering ecology of Loggerhead Shrike has been hindered both by the complexities of tracking movements and an inability to distinguish migrants from year‐round residents during the nonbreeding season, which is a complication for studies of migratory connectivity (see Pérez & Hobson, 2007). Recent advances in the use of genetic and stable isotope markers have greatly improved our ability to quantify migratory connectivity (Hobson and Norris 2008, Rundel et al., 2013; Rushing, Ryder, Saracco, & Marra, 2014). When used together in a Bayesian approach, the two allow precise and accurate delineation of migratory connectivity on an individual‐by‐individual basis (Chabot, Hobson, Van Wilgenburg, McQuat, & Lougheed, 2012). Here, we describe patterns of migratory behavior and quantify the strength of migratory connectivity for Loggerhead Shrikes sampled from across their wintering range using data from genetic and stable isotope markers. We pose several hypotheses as to the ecological, evolutionary, and conservation implications resulting from both patterns and strength of connectivity among breeding populations.

## 2 METHODS

### 2.1 Field data collection

Loggerhead Shrikes were captured using a modified Potter trap, similar to that designed by Craig (1997), which was baited with a live mouse (*Mus mustellus*) held in a protective wire “hardware‐cloth” cage. Sampling occurred from 2004 through 2008. Samples were obtained from both breeding and wintering birds from across the majority of the species range in each season, excluding the northwestern states and Baja peninsula (Figure S1, Table S1). Our sampling scheme targeted 20 individuals per population per season—sample areas in which the species is found year‐round were sampled in both the breeding and wintering seasons. Populations in the northern portion of the species range, where the species is migratory, were sampled only during the breeding season. A few additional areas were sampled only in the wintering season (Figure S1, Table S1).

Shrikes were aged based on the extent of molt in the primary wing feathers (Pyle, 1997), with the exception of sample areas in western Mexico, where only adult birds were sampled and aged simply as After Hatch Year (AHY). Following convention, we refer to any adult bird in their first fall through their first breeding season as Second‐Year (SY) and individuals going into their second or later breeding season as After Second‐Year (ASY) birds.

An outer tail feather was pulled from each bird and a small ~1 cm snip was taken from the distal end of the first primary feather, which is reliably molted on the breeding range (Chabot, Harty, Herkert, & Glass, 2017). Feather samples were used for molecular and stable isotope assays, respectively. Geographic coordinates of capture locations were recorded using a Garmin 12XL hand‐held global positioning system. Breeding season sampling was undertaken between late April and August, and wintering fieldwork occurred from late November through beginning of March, thereby avoiding periods of migration (Yosef, 1996).

For analysis purposes, we divided the nonbreeding range into four regions using available knowledge of the species migration patterns obtained from leg banding data (Burnside 1987). Our regions were also circumscribed by geographic barriers such as mountain ranges, or corresponded to subspecies groupings (Chabot & Lougheed, 2018). Our regions were as follows: Region 1 (Eastern United States), including North and South Carolina, Georgia and Florida; Region 2 (Central United States), including Illinois, Indiana, Tennessee, Missouri, Mississippi, Alabama, Arkansas and Louisiana; Region 3, (Western United States and Eastern Mexico), including Coahuila, Nuevo León, Tamaulipas, Texas, Oklahoma, Colorado, New Mexico and Kansas; and Region 4 (Western Mexico), including sample areas in Chihuahua, Durango, Aguascalientes, Michoacán and Jalisco.

### 2.2 Genetic assays and analysis

Total genomic DNA was extracted from the distal tip of the plucked feather, using a QIAGEN (Venlo, Netherlands) DNEasy Extraction Kit. Fifteen microsatellite loci were assayed, including 14 primer pairs developed for use with Loggerhead Shrike (Coxon, Chabot, Lougheed, Dávila, & White, 2010; Mundy, Winchell, Burr, & Woodruff, 1997) and one developed for the Florida Scrub‐Jay *Aphelocoma coerulescens* but used previously for this species (Mundy et al., 1997). Genetic data obtained from shrikes sampled on the breeding grounds was used to delineate genetic population structure using the Bayesian‐clustering program STRUCTURE 2.3 (Pritchard, Stephens, & Donnelly, 2000). Average admixture coefficients for individuals were derived from the average of 20 runs exported to CLUMPP version 1.1.2 (Jakobsson & Rosenberg, 2007). Geospatial assessment of these genetic signatures suggested 5 genetically and geographically distinct subspecies. A detailed account of the methods and results of the analysis of breeding season genetic population structure can be found in Chabot (2018).

The subset of breeding birds that assigned with 80% or greater probability to a genetic group (Coulon et al. 2008, Fedy et al. 2008) were then used as “trainers” in analysis of the genotypes of birds sampled during the nonbreeding season. The “Use Prior Population Information to Assess Migrants” model in Structure 2.3 (Pritchard et al., 2000) was run with correlated allele frequencies, a burn‐in period of 100,000 replicates and 1,000,000 MCMC iterations and average admixture coefficients were again derived using data from 20 runs exported to CLUMPP v. 1.1.2 (Jakobsson & Rosenberg, 2007).

We assessed connectivity within and among subspecies based on the averaged individual genetic admixture coefficients (*Q*). Individuals were assigned to the subspecies for which *Q* was ≥0.80. Individuals with less than 80% membership in any one subspecies were denoted as “admixed.” Subspecies names followed the terminology of Miller (1931) and Chabot (2018).

### 2.3 Stable isotope assays and analysis

Stable isotope assays were conducted on the snipped primary feather sample following the comparative equilibration method of Wassenaar and Hobson (2003), which uses the precalibrated Environment Canada keratin working standards CBS (−197 ‰), SPK (−121.6 ‰), and KHS (−54.1 ‰). Stable‐hydrogen isotope ratios are reported for the nonexchangeable hydrogen expressed in the typical delta notation (δ^2^H) in units of per ml (‰) normalized on the Vienna Standard Mean Ocean Water‐Standard Light Antarctic Precipitation (VSMOW_SLAP) scale. Based on replicate (*n* = 5) analyses of each standard during analytical runs, measurement error is estimated to be ±2 ‰. Isotope analyses were performed at the stable isotope facility of the National Water Research Centre in Saskatoon, Canada. A detailed account of the methods can be found in Hobson et al. (2014).

Stable isotope (δ^2^H_f_) data obtained from shrikes sampled during the breeding season were supplemented with 40 museum specimens of known breeding provenance from across Mexico obtained from the Universidad Autónoma de Mexico to assist in calibration of the isoscape for Mexico. The isotopic data were then used to derive a species‐specific δ^2^H_f_ isoscape. In brief, we used δ^2^H_f_ data from known‐source (i.e., breeding) shrikes to convert a geospatial model of expected amount‐weighted mean growing‐season δ²H in precipitation (hereafter δ^2^H_p_; Bowen, Wassenaar, & Hobson, 2005) into age‐class specific geospatial models of δ^2^H in Loggerhead Shrike feathers. Based on modeling results, we created separate isoscapes for SY and ASY birds independently. Detailed methods on isoscape development and validation can be found in Chabot et al. (2012).

**Figure 1:**
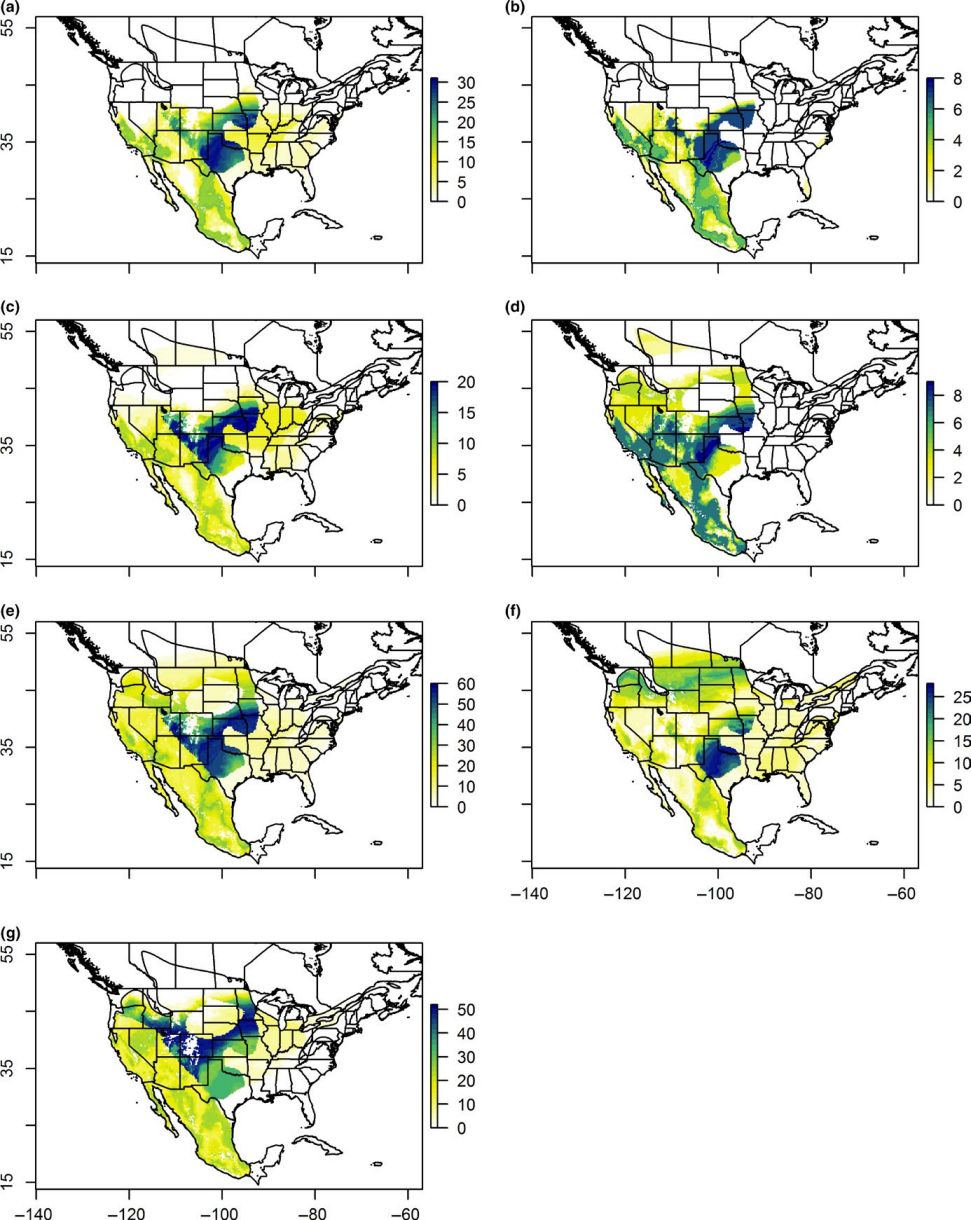
Predicted origins of migratory adult (ASY) and juvenile (SY) Loggerhead Shrike from joint analysis of δ^2^H_f_ and genetic admixture coefficients (*Q*) sampled in (a) the Eastern (ASY), (b) Eastern (SY), (c) Central United States (ASY), d) Central United States (SY), e) Western United States and Eastern Mexico (ASY), f) Western United States and Eastern Mexico (SY), and g) Western Mexico (AHY). *X*‐axis depicts latitude. *Y*‐axis indicates longitude. Bars to right of each graph indicate number of birds in the sample assigned to each pixel

We used the isoscapes and Bayesian assignment methodology developed by Chabot et al. (2012) to assign breeding ground origins to those shrikes sampled during the wintering season. However, as migratory and nonmigratory conspecifics overwinter in the same range, we first had to remove nonmigratory wintering birds before we could investigate patterns of migratory behavior. To do so, we assessed the likelihood that an individual was a local breeder (nonmigratory) by estimating the Bayesian posterior probability that a given individual grew its feather at the sampling location given the individual's δ^2^H_f_ value, conditioned on its genetic admixture coefficient (*Q*); individuals were classified as nonmigratory if the wintering season sample collection location for that individual fell within the region defining the upper 75% of the posterior probabilities of the isoscape. Individuals classified as nonmigrants were removed from further analyses. For each bird identified as a likely migrant, we used normal probability density functions to assess the likelihood that each isoscape pixel in the isoscape represented a potential origin for each individual by comparing observed δ^2^H_f_ against the predicted value; the cells in the isoscape that were consistent with the region defining the upper 75% of the posterior probabilities deemed to be a bird's site of origin. This process resulted in a spatially explicit posterior probability density surface for each individual, which we visualized by graphically depicting the δ^2^H_f_ values for all individuals combined within each of our regions (Figure 1).

We quantified migratory patterns and connectivity in two ways. First, we used general linear models (GLMs) to examine variation in δ^2^H_f_ to determine if individuals exhibited evidence for leap‐frog or chain migration (Salomonsen, 1955), or if shrikes showed differential migration between age classes or subspecies. We restricted our data set to only those areas in which shrikes were aged as SY or ASY and tested all possible models, including sample region, age, and subspecies as independent factors and wintering latitude as a linear covariate. We used Information Theoretic model selection methods based on Akaike's Information Criterion corrected for small sample sizes (AIC_c_, Akaike, 1973; Burnham & Anderson, 1998). Following Burnham and Anderson (1998), models were ranked by second‐order AIC_c_ differences (∆AIC_c_), from which the relative likelihood of each model was estimated. We considered all models with ∆AIC_c_ less than 2.0 from the top model to have strong statistical support (Burnham & Anderson, 1998).

We also assessed the strength of migratory connectivity based on our geospatial assignments to breeding ground origin by calculating the Mantel correlation coefficient (*r*_M_, range ‐1 to 1; sensu Ambrosini, Moller, & Saino, 2009) between pairwise geographic distance matrices of breeding and wintering sites. We estimated the latitude and longitude of the pixel in the isoscape associated the maximum (highest) likelihood of breeding ground origin for each individual and then calculated all pairwise distances between the points for all individuals in the sample area. If more than one isoscape pixel was associated with the highest likelihood value, we calculated mean latitude and longitude since adjacent pixels have similar values. We similarly calculated pairwise distances between wintering ground capture locations. All distances were calculated using great circle calculations (Hobson & Kardynal, 2015). Positive correlations between the distance matrices indicate that individuals that breed more closely together also winter more closely together (Ambrosini et al., 2009).

## 3 RESULTS

**Figure 2:**
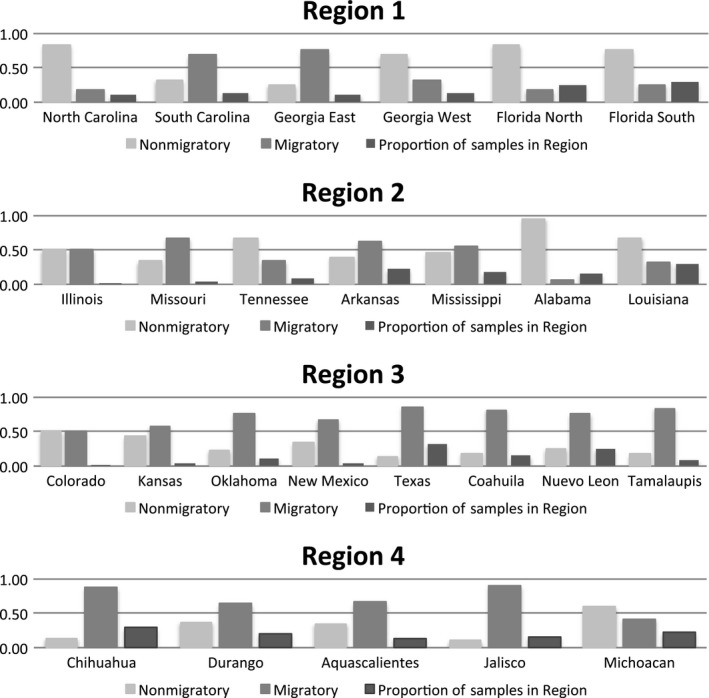
Proportion of individual Loggerhead Shrike assigned as migratory and nonmigratory within each sample area based on genetic admixture coefficient *Q *≥* *0.80 (Eastern United States, Region 1, *n* = 63; Central United States, Region 2, *n* = 54; Western United States and Eastern Mexico, Region 3, *n* = 209, Western Mexico, Region 4, *n* = 130)

In total, we sampled 916 shrike during the nonbreeding season, of which 456 were identified as migrants based on comparison of observed δ^2^H_f_ against the predicted value. The proportion of migrants:nonmigrants varied both among the regions, and among sample areas within each region (Figure 2): 34% (*n *=* *63) of shrikes in the Eastern states (Region 1) were identified as migratory, 40% (*n *=* *54) in Central United States (Region 2), 79% *(n *=* *209) in Western United States/Eastern Mexico (Region 3) and 70% (*n *=* *130) in Western Mexico (Region 4). Sample areas that fell along the Atlantic Coast (Region 1), the Gulf Coast and throughout Texas (Region 3), and throughout Mexico (Regions 3 and 4) supported proportionately more migrants than other sample areas within each region (Figure 2).

Our best model, which included parameters for latitude, age, and region, received 86% of the support among the candidate set of models (Table 1). Parameter estimates from the selected model suggested that δ^2^H_f_ increases from north to south in the wintering grounds (*β* = −1.6 ‰, *SE* = 0.4), implying chain migration. Birds captured in the Atlantic region were substantially more enriched in ^2^H (*β* = 11.8 ‰, *SE* = 2.3) than birds in either East Central United States (*β* = 1.1 ‰, *SE* = 2.7) or Western United States/Eastern Mexico (*β* = 4.4 ‰, *SE* = 12.8). In addition, there was an additive effect of age, suggesting that ASY birds in each nonbreeding area derived from breeding grounds farther south (*β* = 4.1 ‰, *SE* = 1.4) than SY birds captured at the same locale.

**Table 1:** Results of general linear models on spatial patterns of variation in δ^2^H_f_ for migratory Loggerhead Shrike

Model	AIC_*c*_	ΔAIC_*c*_	AIC_*c*_ weight
Latitude+Region+Age	2767	0	0.86
Latitude+Region+Age+Latitude:Region	2772	4	0.11
Latitude+Region	2774	7	0.03
Latitude+Region+Latitude:Region	2778	11	0
Latitude+Subspecies	2779	12	0
Latitude+Subspecies+Latitude:Subspecies	2787	20	0
Latitude+Age	2805	38	0
Latitude+Age+Latitude:Age	2807	40	0
Latitude	2818	51	0
Intercept	2824	57	0

While the distribution of δ^2^H_f_ values suggested migrants from a broad geographic catchment of breeding populations within each region (Figure S2), Mantel tests indicated significant positive migratory connectivity for shrikes in Western United States and throughout Mexico (Regions 3 and 4; Table 2) regardless of age. In contrast, distances between breeding and wintering individuals were uncorrelated for both SY and ASY birds in the Eastern region (*r*_M_ = −0.12, *p *=* *0.75, and *r*_M_ = −0.01, *p *=* *0.53, respectively; Table 2). Second‐Year birds in the Central United States showed no correlation (*r*_M_ = −0.11, *p *=* *0.85), while ASY birds in this area showed weak, marginally nonsignificant positive connectivity (*r*_M_ = 0.16, *p *=* *0.06) (Table 2).

**Table 2:** Results of Mantel's test for connectivity in migratory Loggerhead Shrike based on assigned maximum likelihood of molt origin using δ^2^H_f_ measurements

Wintering ground region	SY			ASY		
	*r*_M_	*n*	*p*	*r*_M_	*n*	*p*
Atlantic Coastal United States	−0.12	14	0.75	−0.01	49	0.53
East Central United States	−0.11	21	0.85	0.16	31	0.06
West Central United States and Eastern Mexico	0.22	77	<0.01	0.07	129	0.02
Western Mexico^a^	–	–	–	0.49	113	<0.01

a Birds aged as After Hatch Year in Mexico included as ASY in analyses.

**Figure 3:**
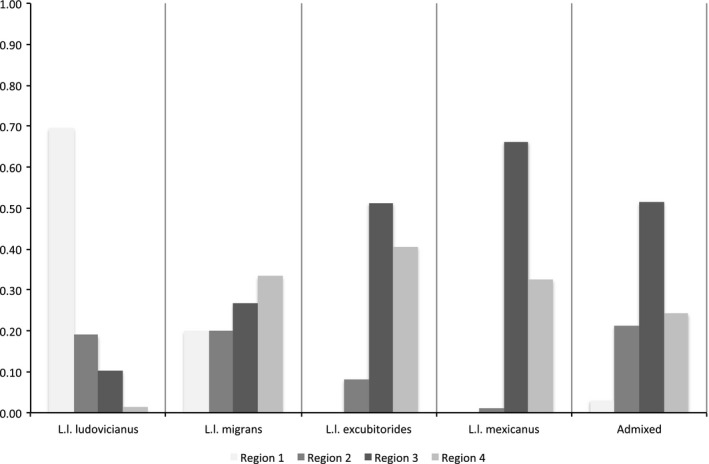
The proportion of winter adults assigned to a subspecies in each nonbreeding region based on admixture coefficients (*Q *≥* *0.80) derived in Structure 2.3 (Pritchard et al. 2000) (Eastern United States, Region 1, *n* = 63; Central United States, Region 2, *n* = 54; Western United States and Eastern Mexico, Region 3, *n* = 209; Western Mexico, Region 4, *n* = 130)

Analysis of genetic admixture coefficients showed differential representation among subspecies within each region (Figure 3). No migratory shrikes were assigned to the newly recognized northeastern subspecies (Chabot 2018), which was not unexpected given its critically small population size (COSEWIC 2014). *Lanius l. migrans*, a partially obligate migratory subspecies, was found throughout the wintering range but in roughly equivalent proportions in each region (Figure 3). *Lanius l. excubitorides*, whose range also falls almost exclusively above 40°N, was found in all regions except the Eastern United States (Region 1), with the majority occurring in Region 3 and 4 (Figure 3). Individuals identified as being migratory and classified as *L. l. ludovicianus*, a putatively nonmigratory subspecies, were found throughout the wintering range, except Western Mexico, with proportions highest in Region 1 (Figure 3). Similar to that, apparently migratory *L. l. mexicanus*, also a putatively nonmigratory subspecies, were also found throughout Regions 3 and 4, but were most common in Western United States and Eastern Mexico (Region 3, Figure 3).

## 4 DISCUSSION

Our study provides the first comprehensive assessment of migratory behavior in the Loggerhead Shrike and is one of the few studies of migratory connectivity combining nuclear genetic and isotopic markers in a Bayesian framework. To the best of our knowledge, ours is also among the first studies of a short‐distance migrant in North America, with overlapping migratory and nonmigratory populations, which complicates assessment of migration. Our analysis relied upon previously developed δ^2^H_f_ isoscapes (Chabot et al., 2012) and genoscape (Chabot 2018).

### 4.1 Migration patterns

While migratory shrikes were present throughout the entire nonbreeding range, our study identified four wintering “hot spots”—the Atlantic Coast, the Mississippi Alluvial Valley, the Gulf Coast, and all of Texas and Mexico. The underlying assumption of hypotheses proposed to explain the segregation of populations across a wintering range is that competitive interactions resulting from density‐dependent factors will constrain the use of the same area by all populations (Alerstam & Högstedt, 1980; Greenwood 1980, Pienkowski, Evans, & Townshend, 1985; Holmgren & Lundberg, 1993). However, our understanding of the conditions leading to patterns of winter distribution among populations is still incomplete. The presence of nonmigratory shrikes in the wintering range of migrants could act as a force against the development of strong migratory linkages between breeding and wintering sites, due to competition for resources unless the two cohorts partitioned the available resources (Tellería & Pérez‐Tris, 2004). Pérez and Hobson (2009) found that wintering habitat use among nonmigratory and migratory Loggerhead Shrikes differed in Mexico, and thus it is likely that this differential use of resources occurs elsewhere in the species’ wintering range. However, the discovery that some wintering areas had higher proportions of migrants, despite the presence of year‐round residents, and strong connectivity in some areas suggests that migration routes and wintering grounds may, in part, be explained by historical range shifts following glaciation, as has been postulated for Swainson's Thrush (*Catharus ustulatus*) (Ruegg & Smith, 2002) and Wilson's Warbler (*Wilsonia pusilla*) (Boulet et al. 2006). Thus, the location of wintering sites for migratory Loggerhead Shrike will be, at least in part, a consequence of the species’ historic range in refugium prior to postglacial expansion (Soltis, Morris, McLachlan, Manos, & Soltis, 2006).

Our data indicate a pattern of chain migration (i.e., the spatial order of migratory populations on the wintering grounds reflects that of the breeding grounds, Salomonsen, 1955), most notably in the western half of the species range. As a result, the northernmost breeders tend to winter farther north than more southerly breeders, thus migratory distance is similar among populations. Our data further suggest a pattern of differential migration of SY and ASY birds. While our broad sampling scheme was not ideal for studying such population‐level processes, the results of other studies of the species support our results. Craig and Chabot (2012) more closely examined the age structure of wintering shrike populations in the Gulf Coast region of eastern Texas. Results indicated fine‐scale patterns of differential migration, with ASY birds preferentially using more coastal sites, and SY birds occurring in more inland areas (Craig & Chabot, 2012). Even in the absence of population‐level differences in migratory behavior, differential migration, in which birds of differing age or sex segregate on the wintering grounds, can create similar patterns to those noted by Salomonsen (1955), thus creating a separate mechanism to reduce competition for resources (Gauthreaux, 1982; Holmgren & Lundberg, 1993; Ketterson & Nolan, 1983; Lundberg & Alerstam, 1986). However, if wintering season threats that are localized, these patterns can result in differential overwintering mortality. Results from long‐term banding studies of the species (Chabot, Hobson, Craig, & Lougheed, 2018) suggest that male shrikes are experiencing higher overwintering mortality rates than females, in particular during their first wintering season. Our results imply a need for further detailed quantification of migration patterns and assessment of overwintering ecology in the species main wintering areas.

### 4.2 Migratory connectivity

Our results indicated differing levels of connectivity occurred among breeding populations of migratory Loggerhead Shrikes on the wintering grounds. Mantel test results indicated connectivity varied from weakly negative in the eastern portion of the species range (Eastern United States SY birds) to strongly positive in more westerly areas (Western Mexico), implying that western individuals both breed and winter more closely together than do eastern shrikes. Comparatively, connectivity was not as strong as that found for Swainson's Thrush (*Catharus ustulatus*;* r*_M_ = 0.72; Cormier, Humple, Gardali, & Seavy, 2013) but, in western populations, connectivity was similar to that found in Montague's Harriers (*Circus pygargus*;* r*_M_ = 0.50, Trierweiler et al., 2016) and Great Reed Warblers (*Acrocephalus arundinaceus*;* r*_M_ = 0.53–0.56, Koleček et al., 2016), and greater than that found for American Wood Thrush (*Hylocichla mustelina*:* r*_M_ = 0.33; Stanley et al., 2015) and European Barn Swallows (*Hirundo rustica*:* r*_M_ = 0.03; Ambrosini et al., 2009). Although variation in connectivity on the order we observed within a single species has not been widely reported, it has been documented in the near‐threatened European Roller (*Coracias garrulous*; Finch et al., 2015) in which western populations exhibited positive connectivity (*r*_M_ = 0.36, *p *=* *0.02) despite no significant connectivity pattern in eastern populations (*r*_M_ = −0.30, *p *=* *0.68).

The strength of migratory connectivity has a deterministic impact on the limiting factors, which may vary within and among populations of the same species (Marra et al., 2011; Norris & Taylor, 2006). While the Loggerhead Shrike has experienced range‐wide declines, regional differences in the trend of breeding population abundance are apparent (Pruitt, 2000; Sauer et al., 2017). Dolman and Sutherland (1994) modeled interactions among habitat loss, population regulation and the evolution of migratory behavior in response to habitat loss on the wintering grounds. They found that, when migratory connectivity was strong, a breeding population was severely affected by habitat loss, but when connectivity was weak, the effect of the loss was reduced. Thus, we speculate that the less severe population declines for western populations, despite higher migratory connectivity, indicates that limiting factors for western populations are not as pronounced as those for eastern populations. Two hypotheses could explain the low connectivity noted for eastern populations, in light of their more persistent and precipitous populations declines. First, limiting factors may be more wide‐spread and/or drastic—for example, habitat loss and degradation may be more severe. Alternatively, given the critically small population sizes of the two eastern subspecies, *L. l. migrans* and *L. l. alvarenis*, our study may simply have not had the power to quantify connectivity for these subspecies, which theoretically could have been high historically. Feather samples obtained from museum specimens could be used to gain a more accurate picture of migratory connectivity, if adequate samples can be obtained. In addition, isotopic analysis of feathers that are reliably molted on the wintering grounds, but obtained from breeding birds, as per the methodology of Greenberg, Marra, and Wooller (2007), could help to quantify migratory connectivity. This approach may prove of particular value for endangered species with small population sizes, in particular for those with broad nonbreeding ranges or nonbreeding ranges that overlap with nonmigratory conspecifics, such as in the Loggerhead Shrike.

An unexpected finding in our study was the assignment of birds identified as migrants based on isotopic values, to *L. l. ludovicianus* and *L. l. mexicanus*, both of which are believed to be nonmigratory subspecies (Yosef, 1996). Our results suggest some individuals undertake long‐distance movements that cannot be attributed to environmental factors. Additional data are needed to differentiate whether these movements represent long‐distance dispersal among breeding areas, or migration‐like “wandering” movements undertaken during the nonbreeding season. The evolutionary consequences will vary based on whether the movements are seasonal “from and to” movements, resulting in limited gene flow that would lead to increasing genetic differentiation over time, or one‐way movements that would facilitate gene flow but limit the rate of local adaptation and speciation (Wright, 1943, 1946). A better understanding of both migratory and dispersal movements are required to develop adequate conservation initiatives. Our results suggest isotopic analysis may be a method by which both can be quantified.

### 4.3 Conservation considerations

Identifying patterns of migratory behavior and nonbreeding season population dynamics remains a high priority, and also a challenge, for most migratory bird species of conservation concern (Elser, 2000; Faaborg et al., 2010; Martin et al., 2007; Runge et al., 2014), in particular, as migratory connectivity impacts the interplay of events throughout the annual cycle (Hostetler et al., 2015). For Loggerhead Shrike, the lack of information on the wintering grounds and wintering ecology has been the greatest obstacle to conservation planning (Cade & Woods, 1997; Pruitt, 2000). Herein, we provide a broad‐scale perspective on migratory patterns that both demonstrate the utility of a Bayesian approach using intrinsic markers and add to the growing body of literature regarding its implications for ecology, evolution, and conservation. We suggest that our methodology is equally suited for broad‐scale and focused smaller scale research. We recommend the latter as the next necessary step in the process whereby limiting factors are identified and conservation plans developed for Loggerhead Shrike. In particular, future research should be focused on the four wintering hotspots we identified and work toward the development of Full Annual Cycle models (reviewed in Hostetler et al., 2015). In the interim, our results will help guide the development of regionally appropriate conservation plans for Loggerhead Shrike that are inclusive of seasons and jurisdictions (sensu Hostetler et al., 2015).

## CONFLICT OF INTEREST

None declared.

## AUTHORS’ CONTRIBUTION

All authors contributed by providing substantial contributions to the conception or design of the work, or the acquisition, analysis, or interpretation of data; drafting or revising the work including providing final approval of the version submitted; and can provide answers to specific parts of the study.
