# Supplementary material for: Migratory connectivity in the Loggerhead Shrike (Lanius ludovicianus)
Source: Ecol Evol. 2018 Oct 24;8(22):10662–72. doi: 10.1002/ece3.4415 (PMC6262747; doi:10.1002/ece3.4415)
Supplement: Supplementary file 1 [file ECE3-8-10662-s001.docx]

Table S1. List of sampling sites included in this study, geographic coordinates bounding the sample area, and number of individuals sampled each season. Breeding season samples from Mexico were obtained from museum specimens.

| Sample locale (Abbreviation) | Season | Latitude (°N) | Longitude (°W) | N _msat_ | N_δ2H_ |
| --- | --- | --- | --- | --- | --- |
| Alabama, US (AL) | B  W | 30.32 to 30.41  30.29 to 30.44 | -87.68 to -87.77  -87.68 to -87.80 | 25  20 | 25  20 |
| Alberta, CND (AB) | B | 49.10 to 51.48 | -110.00 to -112.07 | 52 | 0 |
| Aquascalientes, MX (AQ) | W | 21.64 to 21.81 | -101.96 to -102.30 | 26 | 26 |
| Arkansas, US (AR) | B  W | 34.45 to 34.78  34.53 to 34.70 | -91.87 to -92.17  -91.88 to -92.15 | 22  28 | 22  28 |
| California, US (CA) | B | 33.50 to 38.67 | -117.00 to -122.00 | 39 | 0 |
| Chihuahua-N, MX (CH-N) | W | 30.73 to 31.75 | -108.02 to -108.68 | 27 | 27 |
| Chihuahua-S, MX (CH-S) | W | 20.05 to 29.38 | -101.13 to -106.40 | 27 | 26 |
| Coahuila-N, MX (CU-N) | B  W | 25.28 to 25.38  27.75 to 28.97 | -100.61 to -101.42  -100.51 to -101.03 | 0  18 | 3  18 |
| Coahuila-S, MX (CU-S) | W | 25.19 to 28.73 | -100.71 to -101.02 | 20 | 20 |
| Colorado, US (CO) | B  W | 38.50 to 38.84  38.30 to 38.40 | -104.17 to -105.84  -104.10 to -104.20 | 30  2 | 0  2 |
| Distrito Federal, MX (DF) | B | 19.20 | -99.09 | 0 | 3 |
| Durango, MX (DU) | B  W | 24.02  24.44 to 25.00 | -104.76  -104.68 to -105.09 | 0  45 | 1  38 |
| Florida-NC, US (FL-NC) | W | 29.63 to 29.93 | -82.27 to -82.58 | 22 | 20 |
| Florida-NE, US (FL-NE) | W | 28.77 to 30.25 | -81.11 to -81.60 | 21 | 21 |
| Florida-NW, US (FL-NW) | W | 30.05 to 30.40 | -83.08 to -84.24 | 5 | 5 |
| Florida-SE, US (FL-SE) | W | 25.40 to 25.51 | -80.46 to -80.57 | 26 | 26 |
| Florida-SW, US (FL-SW) | B  W | 27.22 to 27.64  27.20 to 27.45 | -81.60 to -81.87  -81.79 to -82.32 | 24  25 | 23  25 |
| Georgia-E, US (GA-E) | W | 31.93 to 3212 | -81.18 to -81.32 | 20 | 20 |
| Georgia-W, US (GA-W) | B  W | 31.06 to 32.13  31.01 to 32.25 | -83.61 to -83.92  -83.66 to -83.91 | 25  23 | 25  22 |
| Guanajuato, MX (GU) | B | 20.72 to 21.11 | -101.30 to -101.77 | 0 | 3 |
| Guerrero, MX (GE) | B | 17.55 | -99.50 | 0 | 2 |
| Illinois-N, US (IL-N) | B | 41.35 to 41.40 | -88.03 to -88.17 | 10 | 34 |
| Illinois-S, US (IL-S) | B  W | 38.61 to 38.73  37.40 to 38.68 | -87.89 to -88.19  -88.19 to -89.09 | 11  2 | 11  2 |
| Indiana, US (IN) | W | 38.77 | -87.04 | 1 | 0 |
| Iowa, US (IA) | B | 40.71 to 41.43 | -94.37 to -95.17 | 8 | 0 |
| Jalisco, MX (JA) | W | 20.39 to 20.47 | -103.02 to -103.33 | 29 | 29 |
| Kansas-C, US (KS-C) | B | 39.38 to 39.84 | -101.54 to -101.77 | 6 | 0 |
| Kansas-NW, US (KS-NW) | B | 38.61 to 38.67 | -97.76 to -97.94 | 0 | 0 |
| Kansas-SW, US (KS-SW) | B  W | 37.01 to 37.10  37.04 to 37.12 | -100.11 to -100.36  -100.111 to -100.25 | 8  8 | 5  7 |
| Kentucky, US (KY) | B | 36.56 to 36.66 | -86.59 to -86.78 | 10 | 10 |
| Louisiana, US (LA) | B  W | 30.04 to 30.25  30.19 to 30.45 | -92.09 to -92.34  -92.16 to -93.42 | 23  37 | 23  37 |
| Manitoba, CND (MB) | B | 49.67 to 50.15 | -96.75 to -97.65 | 41 | 0 |
| Mexico State, MX (ME) | B | 18.90 to 19.09 | -99.50 to -100.07 | 0 | 3 |
| Michoacan, MX (MI) | B  W | 19.47 to 20.01  19.89 to 20.05 | -101.09 to -101.60  -101.12 to -105.85 | 24  42 | 16  41 |
| Mississippi, US (MS) | B  W | 33.13 to 33.22  33.12 to 38.34 | -90.75 to -91.03  -90.69 to -91.00 | 25  23 | 25  22 |
| Missouri, US (MO) | B  W | 38.13 to 38.29  37.47 to 38.34 | -94.16 to -94.33  -94.19 to -94.38 | 11  6 | 0  6 |
| Montana, US (MT) | B | 47.42 to 48.24 | -104.34 to -105.78 | 10 | 10 |
| Morelos, MX (MR) | B | 18.70 to 18.86 | -98.98 to -99.25 | 0 | 4 |
| Nayarit, MX (NA) | B | 21.39 to 21.55 | -104.83 to -104.86 | 0 | 3 |
| Nebraska, US (NE) | B | 41.17 to 42.93 | -100.50 to -103.67 | 21 | 21 |
| New Mexico, US (NM) | W | 32.00 to 36.28 | -103.27 to -108.00 | 55 | 12 |
| North Carolina-N, US (NC-C) | B  W | 35.32 to 35.82  35.34 to 35.50 | -77.09 to -77.91  -78.70 to -79.05 | 25  18 | 25  18 |
| North Carolina-S, US (NC-S) | B | 34.41 to 34.67 | -78.97 to -79.20 | 25 | 25 |
| North Dakota, US (ND) | B | 46.26 to 47.42 | -102.63 to -103.36 | 20 | 18 |
| Nuevo Leon-E, MX (NL-E) | B  W | 24.71  25.08 to 27.36 | -100.23  -99.01 to -101.02 | 0  55 | 1  55 |
| Nuevo Leon-W, MX (NL-W) | W | 25.71 to 26.01 | -98.88 to -99.62 | 10 | 10 |
| Oaxaca, MX (OA) | B | 17.55 to 17.80 | -97.28 to -97.77 | 0 | 2 |
| Oklahoma-N, US (OK-N) | B  W | 36.23 to 36.97  36.39 to 36.97 | -95.78 to -99.25  -95.48 to -99.15 | 11  13 | 11  6 |
| Oklahoma-S, US (OK-S) | W | 34.18 to 34.49 | -98.07 to -98.23 | 23 | 24 |
| Ontario, CND (ON) | B | 44.32 to 45.47 | -76.65 to -81.13 | 34 | 72 |
| Saskatchewan, CND (SK) | B | 49.10 to 51.94 | -101.91 to -109.81 | 94 | 0 |
| Sinaloa, MX (SI) | B | 25.73 | -107.51 | 0 | 1 |
| South Carolina, US (SC) | B  W | 33.36 to 33.67  33.42 to 33.63 | -80.20 to -80.92  -80.44 to -80.84 | 25  26 | 25  25 |
| South Dakota-N, US (SD-N) | B | 44.36 to 45.20 | -102.28 to -103.13 | 21 | 20 |
| South Dakota-S, US (SD-S) | B | 43.03 to 43.20 | -100.11 to -100.75 | 15 | 14 |
| Tamaulipas, MX (TA) | W | 23.66 to 25.30 | -97.73 to -99.07 | 24 | 24 |
| Tennessee-C, US (TN-C) | W | 35.27 to 35.34 | -87.25 to -87.46 | 12 | 12 |
| Tennessee-W, US (TN-W) | B | 35.63 to 35.99 | -88.94 to -89.32 | 24 | 23 |
| Texas-C, US (TX-C) | W | 31.62 to 31.79 | -96.66 to -96.80 | 24 | 25 |
| Texas-N, US (TX-N) | B | 33.66 to 33.87 | -98.22 to -98.64 | 22 | 22 |
| Texas-S, US (TX-S) | W | 26.15 to 26.50 | -97.63 to -97.82 | 12 | 12 |
| Texas–SE, US (TX-SE) | B  W | 28.66 to 28.98  28.65 to 28.99 | -96.41 to -96.68  -96.41 to -96.69 | 26  42 | 25  49 |


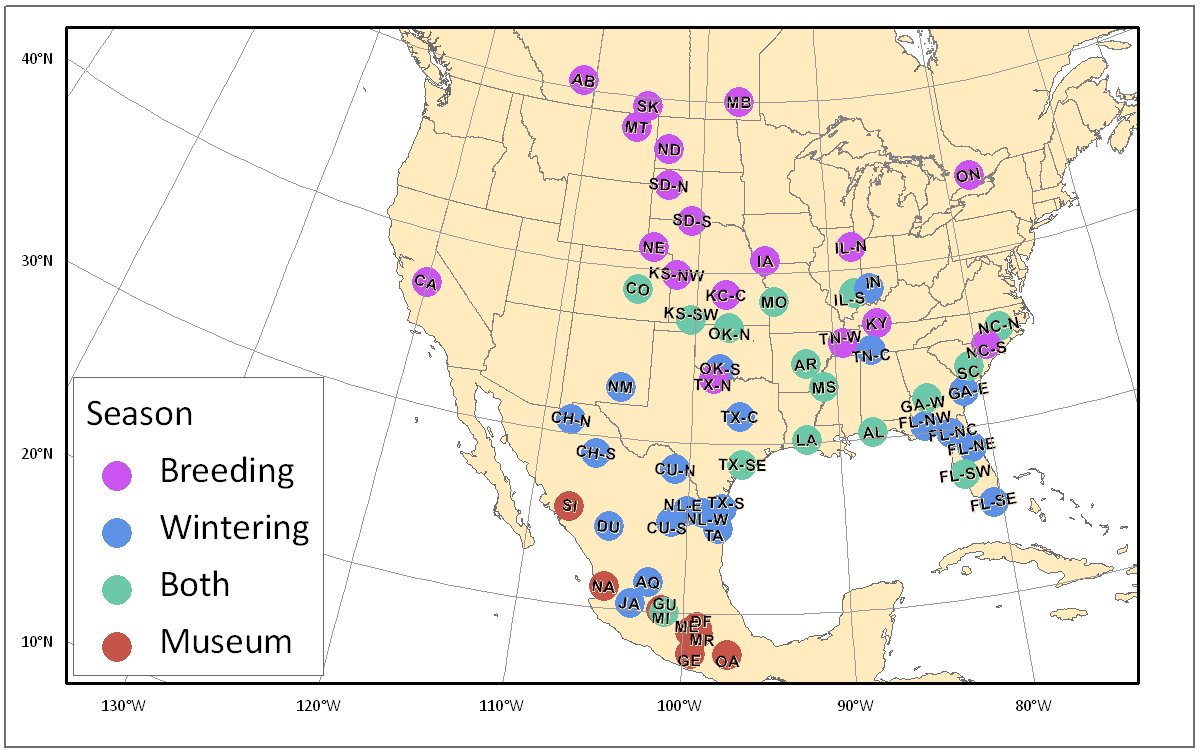


Figure S1. Sampling locales of Loggerhead Shrike feathers used for this study. Abbreviations refer to those in Table 1. Areas denoted as “museum” indicate specimens obtained from museum study skins that were used in creation of the isocapes. “Both” indicates areas in which the population was sampled in during the breeding and nonbreeding seasons. “Breeding” denotes areas only sampled in the breeding season. “Wintering” denotes areas only sampled during the nonbreeding season. See Chabot et al. (2012) for detailed description of isoscape creation and Bayesian assignment methods.

δ^2^H_f_

Figure S2. Box plots of mean and variation in δ^2^H_f_ values among samples within each region (Atlantic, Region 1, n = 63; East Central US, Region 2, n = 54; Western Mexico, Region 4. n = 130; Western US and Eastern Mexico, Region 3, n = 209).
